# NSCLC metastasis: going with ELMO3

**DOI:** 10.18632/oncotarget.2341

**Published:** 2014-08-08

**Authors:** Marie-Anne Goyette, Jean-François Côté

**Affiliations:** IRCM (Université de Montréal) and Department of Anatomy and Cell Biology (McGill), Montréal, Québec, Canada

Lung cancer is the deadliest cancer in the world and 80% of cases are of the non-small cell lung cancer (NSCLC) subtype. Much like other solid cancers, metastatic spread of NSCLC is the primary cause of death for afflicted patients [1]. As such, it is essential to further understand how metastatic progression is orchestrated including the acquisition of invasive characteristics, which are dependent on cytoskeletal rearrangement. The Engulfment and Cell Motility (ELMO) proteins are known to play key roles in cell migration by regulating signaling by the Rho-family member Rac. The association of ELMO with guanine exchange factors of the DOCK-family, including DOCK1, facilitates the relocalization of the complex to discrete areas of the cell membrane where Rac can be activated to promote cytoskeleton remodeling and cell migration [2]. The ELMO family is composed of three proteins: ELMO1, ELMO2 and ELMO3. Previous studies have shown both unique and overlapping functions for ELMO1 and ELMO2 in cell migration, phagocytosis and myoblast fusion [3,4]. In contrast, the biological functions of ELMO3 are poorly defined.

In an article published by Søes et al. in Oncoscience, a role for ELMO3 as a promoter of metastatic dissemination of NSCLC was revealed [5]. The authors studied the DNA methylation status of the *ELMO3* promoter and the expression levels of *ELMO3* mRNA in 26 human NSCLC samples. They determined that *ELMO3* expression was higher in primary tumors from patients with metastasis, as well as in their matched metastases, in comparison to normal lung tissue and primary tumors of patients without metastasis. The overexpression of *ELMO3* coincided with hypomethylation of its promoter. Are the cancer cells expressing high level of *ELMO3* in the primary tumors enriched at the metastasic sites? This does not appear to be the case as no significant difference in the expression of *ELMO3*, or the methylation status of its promoter, was found between the primary tumors and matched metastases of patients. Because mRNA and protein levels are not always correlated, it will be important in the future to analyze ELMO3 expression at the protein level in primary tumors and metastases. Nevertheless, high expression of *ELMO3* is likely to confer an advantage to tumor cells to reach distant sites including brain and adrenal glands.

All three members of the ELMO family share similar modular protein domains [1], but ELMO3 differs the most when comparing protein sequences. While ELMO3 likely binds DOCK exchange factors much like ELMO1/2, it may possess a unique set of interacting partners present in NSCLC cells that could be involved in promoting metastasis. Future studies will be required to define the molecular mechanisms whereby ELMO3 promotes cytoskeleton remodeling.

The microRNA *miR-328* is located in the same locus as the *ELMO3* gene. *miR-328* expression was found deregulated in NSCLC cell lines such that its expression promotes migration [6]. Moreover, *miR-328* is also proposed as a blood biomarker for diagnostic of NSCLC patients at high risk of developing brain metastasis [7]. However, Søes et al. found that *ELMO3* and *miR-328* expression are inversely correlated such that *miR-328* is less expressed in primary tumors compared with normal tissue. This result therefore suggests that using this microRNA as a biomarker will not be possible.

Moving forward, functional experiments should be carried out in order to understand the mechanism of action of ELMO3 in the formation of metastasis in NSCLC. *In vitro* assays could be done in NSCLC cell lines to confirm if ELMO3 is a key player in migration. In addition, interacting partners that link ELMO3 to cytoskeletal rearrangement should be determined. I*n vivo* assays would strengthen the connection between ELMO3 expression and metastasis in NSCLC. Experimental metastasis assays, such as tail vein injection of NSCLC cells with decreased ELMO3 expression, would provide valuable information. When generated, ELMO3 KO mice could be crossed with a transgenic mouse model of lung cancer to validate the role of ELMO3 in metastasis in a model reflective of the human disease [1]. Finally, the correlation with *miR-328* could be tested experimentally. For example, could forced expression of this microRNA decrease ELMO3 expression and migration in cell systems?

In conclusion, this study by Søes et al. reveals *ELMO3* as a possible key player in metastasis. This finding may lead to a better understanding of the metastatic process in NSCLC progression. Importantly, it identifies a new therapeutic target for lung cancer for the treatment of this deadly disease.
